# Parkinson’s Disease Medication Adherence Scale: Conceptualization, Scale Development, and Clinimetric Testing Plan

**DOI:** 10.3389/fnagi.2022.900029

**Published:** 2022-05-13

**Authors:** Michelle H. S. Tosin, Christopher G. Goetz, Dharah P. C. F. Bispo, Henrique B. Ferraz, Marco Antonio A. Leite, Deborah A. Hall, Glenn T. Stebbins, Beatriz Guitton R. B. Oliveira

**Affiliations:** ^1^Department of Nursing, Fluminense Federal University, Niteroi, Brazil; ^2^Department of Neurological Sciences, Rush University Medical Center, Chicago, IL, United States; ^3^Department of Neuropsychiatry and Behavioral Sciences, Federal University of Pernambuco, Recife, Brazil; ^4^Department of Neurology, Federal University of São Paulo, São Paulo, Brazil; ^5^Department of Neurology, Fluminense Federal University, Niteroi, Brazil

**Keywords:** scale development, measurement, psychometrics (MeSH), medication adherence (MeSH), Parkinson’s disease

## Abstract

**Background:**

Medication adherence is a crucial component in the management of patients with chronic diseases needing a long-term pharmacotherapy. Parkinson’s disease (PD) is a chronic, degenerative disease with complex drug treatment that poses challenging barriers to patient adherence. The adoption of best practices of scale development can contribute to generate solid concepts and, in the long run, a more stable knowledge base on the underlying constructs of medication adherence in PD measured by the items of the first scale to be created for this purpose.

**Purpose:**

To present the development process and clinimetric testing plan of the Parkinson’s Disease Medication Adherence Scale (PD-MAS).

**Method:**

We adopted a hybrid approach plan based on the United States Food and Drug Administration and Benson and Clark Guide that will create a patient-reported outcome instrument. We presented an overview of consecutive and interrelated steps, containing a concise description of each one. International research centers from Brazil and United States were initially involved in the planning and implementation of the methodological steps of this study.

**Results:**

We developed a four-phase multimethod approach for the conceptualization and the clinimetric testing plan of the PD-MAS. First, we describe the development process of the conceptual framework of the PD-MAS underpinning the scale construct; second, we formalized the development process of the first version of the PD-MAS from the generation of item pools to the content validation and pre-testing; third, we established the steps for the first pilot testing and revision; fourth, we describe the steps plan for the first pilot testing and revision, to finally describe its clinimetric testing plan and validation.

**Conclusion:**

The overview presentation of the development phases and the clinimetric testing plan of the PD-MAS demonstrate the feasibility of creating an instrument to measure the multidimensional and multifactorial components of the medication adherence process in people with PD.

## Introduction

Despite increased awareness, suboptimal medication adherence among people treating chronic diseases remains a global problem (Sabaté, 2003). Among people with Parkinson’s disease (PD), suboptimal adherence to medications ranges from 10 to 67% (Davis et al., 2010), and is currently measured using two methods: 1. direct, defined as objective measurements of concentrations of the medication or its metabolites (e.g., measurement of drug and metabolite levels in the blood and/or urine); and 2. indirect, defined as subjective measures of proxies’ observations (e.g., pill count, electronic monitoring devices, electronic health records, and rating scales) (Grosset et al., 2006; Kulkarni et al., 2008; Davis et al., 2010). So far, there is no standard procedure for measuring medication adherence, and both methods have advantages and disadvantages. In people with PD, the use of rating scales to accurately measure the behaviors influencing the medication adherence process may have additional disadvantages, because of the absence of an instrument developed specifically to measure this construct in this population (Lam and Fresco, 2015).

To date, the only rating scale created to assess medication adherence in PD, measures only one domain of this construct: the belief that people with PD have regarding their antiparkinsonian medications. Developed in 2016, the instrument “Parkinson’s Disease Medication Beliefs Scale (PD-Rx)” was tested by scientists in a first pilot study. The scale aims to identify the beliefs that underlie drug phobia. However, in a pilot test of the clinimetric properties of the measure, the authors emphasize that the study had limitations related to both the sample (small and homogeneous), and the lack of more robust measures to test convergent validity (e.g., electronic drug monitoring devices) (Fleisher et al., 2016).

The challenge of developing and validating a rating scale to measure medication adherence specifically in people with PD, depends on the ability of this instrument to capture, in a reliable and valid way, the set of dimensions and factors involved in the medication adherence process, assembling the most appropriate items to constitute test questions. For example, the presence of non-motor symptoms of PD, such as cognitive impairment, depression, apathy, excessive daytime sleepiness, or concomitant psychosis, has been shown to influence adherence (Mendorf et al., 2020). Likewise, the use of different drug presentations (such as oral versus patch medications, fractionated versus single-dose medications) has been shown to be factors involved in the medication adherence process in this population (Mynors et al., 2007; Schnitzler et al., 2010). To capture these and other factors that could potentially influence adherence, it is recommended that a comprehensive measuring instrument follow an inclusive approach. This requires the scale developers to have specific theoretical, methodological, and statistical competencies in clinimetrics, as it involves the collection and analysis of primary and secondary data (Boateng et al., 2018). These competencies are not usually taught but are duplicated from procedures reported in scientific papers (Carpenter, 2018).

The proper construction of a scale has important implications for the inferences of the measure, as it affects both the quality and size of the effects obtained and the statistical significance of these effects, reflecting the precision and sensitivity of the instrument (Furr, 2018; Kyriazos and Stalikas, 2018). Precise measurements of the severity and impact of PD symptoms and response to drug treatment are necessary for the development of symptomatic and disease-modifying therapies (Espay et al., 2017; Pires et al., 2017; Paolini Paoletti et al., 2020) and the current literature is vast in evidence on clinical findings and their impacts in PD patients (Tan et al., 2014; Muzerengi et al., 2016; Kramer et al., 2018). However, studies that correlate these data to medication adherence, as measured using scales, are scarce, raising the need to create a rating scale that measures medication adherence specifically for the context of PD.

To produce more solid concepts in the long term with a more stable knowledge base on adherence to PD medication, in this study protocol, we followed the standard recommendations of the instrument developers (Benson and Clark, 1982; US Food and Drug Administration, 2009; Speight and Barendse, 2010), and outline a clear and objective design of the steps for the development and validation of the Parkinson’s Disease Medication Adherence Scale (PD-MAS).

## Methods

### Study Design

**FIGURE 1:**
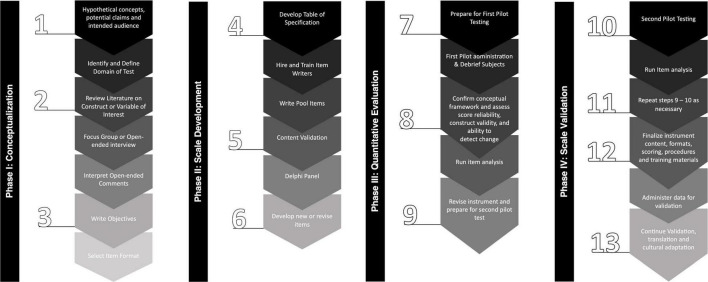
Illustrative overview of a scale development and validation process.

This is a clinimetric study protocol for rating scale development. We adopted the United States Food and Drug Administration (FDA) (Benson and Clark, 1982; US Food and Drug Administration, 2009) and Speight and Barendse (2010) guidance for developing a patient-reported outcome (PRO) instrument for use as a clinical trial. This guidance met both best practices for developing and validating rating scales for health, social, and behavioral research (Boateng et al., 2018), and the recommendations of the International Parkinson and Movement Disorders Society (MDS) Task Force on Rating Scales in Parkinson’s Disease: clinical practice and research (Sampaio et al., 2012; Figure 1).

### Study Settings

International movement disorders outpatient clinics of the following university hospitals have been initially involved in the planning for future implementation of the methodological steps of this study: Antônio Pedro Hospital (HUAP), of the Fluminense Federal University (UFF), Niterói, Brazil; São Paulo Hospital, of the São Paulo Federal University (UNIFESP), São Paulo, Brazil; and Rush University Medical Center (RUMC), Chicago, IL, United States. These are tertiary centers specialized in the care of patients with movement disorders, including PD, with neurologists, nurses, ancillary health professionals, and a multidisciplinary team specialized in this area.

As these centers have different languages (Portuguese and English), the PD-MAS will be created in these two language versions, complying with parsimony criteria between the two versions.

## Results

The PD-MAS development process was designed to be implemented in four phases, including: (1) conceptualization; (2) scale development; (3) quantitative evaluation; and (4) validation. Each phase has interactive steps with a multimethod approach for the collection and analysis of primary and secondary data that will support the integral development of the PD-MAS. The aims and activities that will be carried out in each phase of the development of the PD-MAS are summarized in Supplementary Table 1.

### Phase I: Conceptualization Overview

The conceptual framework basis of the PD-MAS will be determined following Delphi panel of experts based on the population of interest; objectives of the instrument; global domain; content areas; questions to be answered; purposes of the measurement items; and the indications for its use. Then, to adequately address the observable and relevant phenomena of the global domain and subdomains addressed by the PD-MAS, primary and secondary sources of information will be explored.

As a secondary source, a non-systematic review of the literature on the topic “medication adherence,” “Parkinson’s disease,” and “medication adherence in people with PD” will provide a preliminary conceptual definition of the domain, and provide a preliminary assessment of the medication adherence scales used in studies with people with PD. Following this non-systematic review, a systematic review of the literature will identify the domains and subdomains assessed by the medication adherence rating scales used in studies with people with PD, and whether the essential components of medication adherence are adequately covered.

As a primary source, focus groups with people with PD and their caregivers will be conducted, and observable and relevant phenomena of medication adherence that may not have been identified in the secondary source will be captured. Given the heterogeneity of PD disabilities and impairments, such focus groups will need to cross different age groups, disease stages, family structures, cultural, financial, and health system differences.

Lastly, the data from both secondary and primary sources will be combined through data triangulation to allow quantitative and qualitative analysis that will establish the specific measurement objectives of the PD-MAS, that will form the basis of the preliminary scale development phase.

### Phase II: Scale Development Overview

From the objectives of the PD-MAS obtained in the previous phase, we will elaborate the items that will constitute the PRO, considering the criteria determined for its format and scoring model (Benson and Clark, 1982). As recommended, we will develop an exhaustive number of items to ensure that enough for the final version of the scale (Natalio et al., 2014). The excessive creation of items will ensure that enough items are obtained for the final version of the scale without the need to revisit the previous steps at a more advanced stage (Natalio et al., 2014).

Subsequently, a careful review of each item will be conducted according to pre-established criteria and will be checked if: they are clearly defined; they meet the selected format; the answer options are plausible and excluding; the terms used are adequate; the wording is clear; the phenomena identified in the three data sources are considered; they are consistent with their conceptual definitions; and they are representative and relevant to the global domain and the clinical interpretation (Benson and Clark, 1982; Natalio et al., 2014). The selected item will form the first prototype of the PD-MAS will be established. This phase will be concluded after a content validation from a Delphi panel of specialists and a cognitive pretesting with people with PD, their caregivers and movement disorders specialists. The cognitive pretesting will examine the extent to which the questions reflect the domain under study, and the extent to which the respondent feels comfortable with the response options and feels that the response options are appropriate and if answers to the questions produce valid measurements that are meaningful to the patient and/or caregiver.

We anticipate that the reporting source of information for the PD-MAS will need to be flexible to accommodate the patient and a primary caregiver. As such, we envision two validated versions, “PD-MAS: Patient” and “PD-MAS”: caregiver. In cases where the management and administration of medication for PD is done in cooperation between the person with PD and their caregiver, both must respond to the scale independently, each using the most appropriate version of the PD-MAS.

### Phase III: Quantitative Evaluation Overview

The testing plan of the PD-MAS is composed by sequential clinimetric phases of quantitative evaluation of the scale’s measurement properties using Classical Test Theory (CTT) (DeVellis, 2006) and Item Response Theory Analysis (IRT) (Hays et al., 2000). Through large international cross-sectional study with people with PD, the measurement properties of the scale will be tested according to the follow criteria: reliability, structural validity, internal consistency, measurement error, criterion validity, hypotheses testing for construct validity and responsiveness of the scale. This phase of testing will entail healthcare professionals directly involved in the care of people with PD, and they will be trained to use the PD-MAS in their clinical and research practices. They will also be trained in entering data in the database that will be created specifically to store the data from these phases of the study. Demographic information about each patient who participates in these phases will identify profiles of people with PD who will predict the need to gather the questionnaire data through an interview based on evaluators. The added rater time will be calculated and incorporated into the clinimetric feasibility analysis. The version of the scale that meets the criteria of sufficient reliability, validity and responsiveness and accommodates maximum information without duplication will become PD-MAS in its final form.

### Phase IV: Scale Validation Overview

It is possible that a second pilot testing will be done to fill in the gaps identified in the first pilot testing. Should it be required, the data will be analyzed again, and as necessary, some phases may be resumed as the version of the scale needs to meet the criteria of sufficient reliability, validity, and responsiveness. This process may be iterative and is designed to allow the scale to capture the maximum amount of unduplicated information in the final PD-MAS form.

From here, we intend not to restrict ourselves to the English and Portuguese versions only, but to translate the PD-MAS into other languages. Therefore, the translated versions will be submitted to a responsivity testing program for PD-MAS, as well as tested through qualitative cognitive assessments in a small number of patients for each language under consideration.

At the conclusion of each phase, we will assess the need for revisions of the PD-MAS. We recognize that the scale may in fact evolve over the phases of clinimetric testing and, therefore, we will not publish any draft before its final version. However, we anticipate that future projects offer an opportunity for individual researchers, societies, and industries to be part of the studies that will validate the first scale created to measure the multidimensional and multifactorial components involved in the medication adherence process in patients with PD.

## Discussion

Despite the availability of literature on the theory of development and validation of scales (Benson and Clark, 1982; McDowell, 2006; Stebbins, 2012; Boateng et al., 2018; Carpenter, 2018; Kyriazos and Stalikas, 2018), the conceptualization, design, clinimetric testing, and validation analysis of a new scale need to be done with a sensitivity to scientific rigor and practicality related to the population of interest, in this case, patients with PD.

According to COSMIN (COnsensus-based Standards for the selection of health Measurement INstruments), it is important that clinicians and researchers analyze at least six clinimetric criteria before selecting health measurement instruments, including: (1) a description of the conceptual framework that explains which concepts are being measured by scale; (2) what is the target population and their health condition; (3) how items should be weighted according to each of the scale’s subdomains; (4) what is the mode of administration and data collection; (5) what are the response options and their appropriate scores; and finally, (6) the possibility of translation or cultural adaptation (Mokkink et al., 2018). To meet these criteria, this research devoted considerable resources of time to describe the development process and clinimetric testing plan of the PD-MAS, providing the international community with official definitions and technical procedures that will be used in all phases of development of this rating scale.

Because the development and validation phases will be conducted in multinational settings, including raters, patients and caregivers who are not native-English/Portuguese speaking, this study protocol provides an assurance of homogeneity in the implementation of research procedures. Similar initiatives have been used in clinimetric studies in PD, and with this infrastructure work before the field tests, the scales that were finally tested on a large scale were successful, emphasizing the relevance that this methodological rigor has before the start of scale development (Goetz et al., 2014; Comella et al., 2015).

Finally, we expect that the information resulting from longitudinal research data on medication adherence in PD can be used to support disease-based outcomes and additional impacts on patients and their care partners, such as: quality of life, burden, functional level, levodopa equivalent daily dose (LEDD) and adverse effects. Furthermore, we expect that the data from the PD-MAS will support the results of studies that express other clinical indices, such as those related to motor and non-motor symptoms of PD.

## Conclusion

As science advances and novel research questions are put forth, new scales become necessary. The use of multiple items to measure an underlying latent construct can account for, and isolate, item-specific measurement error, which leads to more accurate research findings. The development of a new scale is a challenging process given the involvement of several theoretical, methodological, and statistical competencies.

The overview presentation of the development phases and the clinimetric testing plan of the PD-MAS demonstrate the feasibility of creating an instrument to measure the multidimensional and multifactorial components of the medication adherence process in people with PD. With this report, researchers will be equipped for the proper implementation of the techniques of this long-term study.

## Author Contributions

All authors contributed to the conception and design of the study. MT, BO, and GS organized the database and the statistical analysis plan. MT wrote the first draft of the manuscript. All authors contributed to writing the manuscript, reviewing the statistical plan, and approving the submitted version.

## Conflict of Interest

The authors declare that the research was conducted in the absence of any commercial or financial relationships that could be construed as a potential conflict of interest.

## Publisher’s Note

All claims expressed in this article are solely those of the authors and do not necessarily represent those of their affiliated organizations, or those of the publisher, the editors and the reviewers. Any product that may be evaluated in this article, or claim that may be made by its manufacturer, is not guaranteed or endorsed by the publisher.
