# Supplementary material for: Parkinson’s Disease Medication Adherence Scale: Conceptualization, Scale Development, and Clinimetric Testing Plan
Source: Front Aging Neurosci. 2022 May 13;14:900029. doi: 10.3389/fnagi.2022.900029 (PMC9136049; doi:10.3389/fnagi.2022.900029)
Supplement: Supplementary file 1 [file Table_1.DOCX]

Supplementary Material

# Supplementary material 1. The four phases and thirteen steps of PD-MAS development and validation.

| **Phases & Steps** | | **Aims** | **Method & Procedures for Data Analyses** | |
| --- | --- | --- | --- | --- |
| ***Phase I: Conceptualization*** | | | | |
| **1** | Hypothetical concepts, potential claims and intended audience | To determine the conceptual basis of the PD-MAS. | Qualitative approach  The team will discuss the construct to define the conceptual basis of the PD-MAS. | |
|  | Identify and define domain of test | To identify and define the PD-MAS domain using different sources of information. |  |  |
| **2** | Review the literature on the construct or variable of interest | To identify the content available in the current literature related to the theme. | Quantitative and qualitative approach   - Secondary source - Scientific literature   - Non-systematic review   - Systematic review (PRISMA) - Primary source – Focus groups with patients and caregivers | Data triangulation |
|  | Create and apply open-ended questions to the focus group | To understand the perception of the focal group regarding the proposed theme. |  |  |
|  | Interpret open-ended comments | To analyze the results from these 3 sources. | Qualitative approach   - Computer-assisted analysis: Similarity analysis and descending hierarchical classification - Critical analysis of narrations: codification of PD-MAS subdomains |  |
| **3** | Write objectives | To determine the objective of the PD-MAS. | Qualitative approach  The team will discuss and establish the specific measurement objectives of the PD-MAS. It will be documented preliminary instrument development. | |
|  | Select item format | To define the best format for the PD-MAS. |  |  |
| ***Phase II: Scale Development*** | | | | |
| **4** | Develop table of specification | To elaborate the items that will constitute the PD-MAS. | Qualitative approach  • Excessive item creation  • Careful review of each item  • Item set review | |
|  | Hire and train item writers |  |  |  |
|  | Write pool items |  |  |  |
| **5** | Content validation (Delphi panel) | To validate the content of the PD-MAS. | Quantitative and qualitative approach – Delphi panel of specialists:  Each item will be reviewed and checked. The 1^st^ prototype of the PD-MAS will be established, and its development will be concluded after a content validation, using CVR. | |
|  | Cognitive pretesting | To evaluate whether the items reflect the domain of study and meet the requisite standards | Qualitative approach – Cognitive interviews   - ﻿Administer draft items to 5 to 15 patients with PD, their caregivers and movement disorders specialists in 2 to 3 rounds assessing their mental process entailed in providing answers. - Analyze the qualitative data coming from the comments and suggestions provided by the participants - Generate recommendations for changes in the items - Revise the items between rounds until reach data saturation and no further adjustments are needed | |
| **6** | Develop new or revise items | To review the last steps. | Qualitative approach  The team will review the items. | |
| ***Phase III: Quantitative Evaluation*** | | | | |
| **7** | Prepare instrument for first pilot testing | To verify that the proposed theme is being covered in the PD-MAS. | Prior to the pilot test, the revised items will be typed to produce the test form. | |
|  | First pilot testing & Debrief subjects | To establish the dimensionality, feasibility, and acceptability, of PD-MAS to assess medication adherence behaviors in PD | Collect data cross-sectionally and then, analyze them according to the following measurement procedures:   - Item reduction: exploratory factor analysis (inter-item and item-total correlations) using R   - Determine the factorability of the data measuring sampling adequacy using the KMO ≥ 60 - Extraction of factors: factor analyses determining the optimal number of domains of medication adherence to the latent measure of the disease, as measured by the PD-MAS. - 1PL & 2PL of the IRT modeling, run on the mirt program implemented in R. - Tests of dimensionality: confirmatory factor analysis to test the dimensionality of the scale.   - Lavaan approach available in R to determine the CFI ≥ 0.95 | |
| **8** | Confirm conceptual framework and assess score reliability, construct validity, and ability to detect change | To verify the reliability, validity, and responsiveness of the PD-MAS. | - Test the potential scale items in a heterogeneous sample that reflects and captures the range of the target population - Use the rule of thumb of at least 10 participants for each item on the scale - Tests of reliability:   - Cronbach’s alpha ≥0.80.   - ICC: where the higher the correlation, the higher the test–retest reliability (≥0.70) - Tests of validity: Examine baseline differences in the outcome measures between PD patients and caregivers   - ANOVA or ANCOVA with appropriate covariates - Sensitivity to change: General linear model of PD patients and caregivers’ samples with a minimal effect size of d > 0.5 to indicate sensitivity to change   - MID: calculated using anchor (MCID) and distribution-based methods. | |
|  | Run item analysis |  |  |  |
| **9** | Revise instrument and prepare for second pilot test | To review the information collected and verify if the PD-MAS is parsimonious (item reduction). | The necessary changes will be made based on the analysis carried out and it will be documented the measurement of the development of the PD-MAS. | |
| ***Phase IV: Scale Validation*** | | | | |
| **10** | Second pilot testing | To verify the reliability, validity of the second version. | Quantitative approach  Same approach as step 8 | |
|  | Run item analysis | To analyze the information that was collected. | Qualitative approach  Same approach as step 9 | |
| **11** | Repeat steps 9 – 10 as necessary | To improve the items that need improvement if it is necessary. | Qualitative approach  This step can happen when the research needs to provide more adjustments to this scale. | |
| **12** | Finalize instrument content, formats, scoring, procedures, and training materials | To establish the final version of the PD-MAS. | Qualitative approach  The version of the scale that meets will checked based on specific criteria. | |
|  | Administer data for validation | To determine the data for validation. | Qualitative approach  Content validation (Delphi panel) will be repeated with the final version. | |
| **13** | Continue validation, translation, and cultural adaptation | To execute the validation, translation, and cultural adaptation. | It will intend to outline a translation and back-translation to non-English and non-Portuguese languages. | |

**Legends:** PD-MAS: Parkinson’s disease Medication Adherence Scale | CVR: Content Validity Ratio | PRISMA: preferred reporting items for systematic reviews and meta-analysis | PD: Parkinson’s disease | R statistical program: R Foundation for Statistical Computing, Vienna, Austria | KMO: Kaiser-Meyer-Olkin | 1PL: One-parameter logistic model | 2PL: Two-parameters logistic model | IRT: Item Response Theory | CFI: Comparative Fit Index | ICC: Intra Class Correlation Coefficient | ANOVA: Analysis of Variance | ANCOVA: Analysis of Covariance Models | MID: Minimal important difference | MICD: minimal clinically important difference.
